# CDK4/6 Inhibitors Overcome Endocrine ESR1 Mutation-Related Resistance in Metastatic Breast Cancer Patients

**DOI:** 10.3390/cancers15041306

**Published:** 2023-02-18

**Authors:** Stefania Crucitta, Martina Ruglioni, Giulia Lorenzini, Irene Bargagna, Giovanna Irene Luculli, Irene Albanese, Diana Bilancio, Francesca Patanè, Andrea Fontana, Romano Danesi, Marzia Del Re

**Affiliations:** 1 Unit of Clinical Pharmacology and Pharmacogenetics, Department of Clinical and Experimental Medicine, University of Pisa, 56126 Pisa, Italy; 2 Unit of Medical Oncology, Department of Translational Research and New Technologies in Medicine, University of Pisa, 56126 Pisa, Italy

**Keywords:** ESR1 mutation, liquid biopsy, ctDNA, metastatic breast cancer, CDK4/6 inhibitor

## Abstract

**Simple Summary:**

The present study investigates the clinical benefit of CDK4/6i in ESR1 mutant HR+ mBC patients treated with a CDK4/6i as first- or second-line therapy. Plasma was collected at baseline prior to CDK4/6i plus hormone therapy, and ESR1 mutation was analyzed in circulating free DNA by a ddPCR. This study demonstrates that the ESR1 mutations detected in liquid biopsy is an independent predictive factor of clinical recurrence in the adjuvant setting. No difference in progression-free survival (PFS) was observed in the presence or absence of ESR1 mutations in patients treated with CDK4/6i as first-line treatment. The results suggest that CDK4/6i can overcome ESR1-dependent resistance.

**Abstract:**

ESR1 mutations contribute to endocrine resistance and occur in a high percentage of hormone-receptor-positive (HR+) metastatic breast cancer (mBC) cases. Cyclin-dependent kinase 4/6 inhibitors (CDK4/6i) changed the treatment landscape of HR+ mBC, as they are able to overcome estrogen resistance. The present retrospective study investigates the clinical benefit of CDK4/6i in ESR1 mutant HR+ mBC patients treated with a CDK4/6i as first- or second-line therapy. Plasma was collected at baseline prior to CDK4/6i plus hormone therapy as a first- or second-line treatment. Circulating free DNA (cfDNA) was extracted from plasma, and ESR1 mutation analysis was performed on a ddPCR. Statistical analyses were performed to investigate the predictive power of ESR1 mutations and any association with clinical factors. A total of 42 patients with mBC treated with CDK4/6i plus endocrine therapy as first- (*n* = 35) or second-line (*n* = 7) were enrolled. Twenty-eight patients received hormonal therapy (AI or tamoxifen) in the adjuvant setting. ESR1 mutation status in blood was associated with shorter median disease-free survival (DFS) (30 vs. 110 months; *p* = 0.006). Multivariate analysis confirmed ESR1 mutations as independent factors of resistance in adjuvant hormone therapy. On the contrary, no difference in progression-free survival (PFS) was observed in the presence or absence of an ESR1 mutation in patients treated with CDK4/6i as first-line treatment (*p* = 0.29). No statistically significant correlation between the best response to CDK4/6i and ESR1 mutation was found (*p* = 0.46). This study indicates that the ESR1 mutation detected in cfDNA is an independent predictive factor of clinical recurrence in the adjuvant setting and that CDK4/6i can overcome ESR1-dependent resistance.

## 1. Introduction

Breast cancer is characterized by a high level of molecular heterogeneity, which is one of the main causes of resistance to therapies due to the adaptation of cell clones under the selective pressure of treatments, leading to the acquisition of new resistance alterations [1]. Almost 70% of breast cancers are hormone-receptor-positive (HR+), and in these patients endocrine therapy is the backbone of treatments [2]. However, resistance inevitably occurs and, among some patients, may be associated with mutations within the ligand-binding domain (LBD) of the estrogen receptor-1 (ESR1) gene [3,4]. The LBD is considered a ‘hotspot’ region that promotes tumor growth, potentially enhancing treatment resistance, leading to the constitutive ligand-independent ER activation [3,5]. The most common ESR1 point mutations are present in codons 537 and 538, followed by others that have been identified with lower frequencies [3,6]. Data report that the prevalence of the ESR1 mutations depends on the duration and setting of the endocrine therapy and that they seem to occur almost exclusively after aromatase inhibitors in metastatic breast cancer (mBC) patients [7,8,9]. Recent data highlighted the potential role of ESR1 mutational status as a predictive biomarker and a tool to guide clinicians in therapeutic decisions [7,10]. Recently, new therapeutic strategies have been developed, including cyclin-dependent kinase 4/6 inhibitors (CDK4/6i). Interestingly, it is known that ESR1 mutations respond differently to treatment due to the polyclonal origin of such ESR1 variants, in addition to high tumor molecular heterogeneity [11]. Moreover, the detection of ESR1 mutations has been associated with clinically inferior outcomes, including progression-free survival (PFS) and overall survival (OS), in comparison to non-mutant ESR1 patients treated with exemestane plus everolimus [12]. Recent data demonstrated that the use of CDK4/6i may overcome treatment resistance to hormonal therapies, allowing for prolonged survival in the metastatic setting [13,14]. Tracking ESR1 mutations through the use of circulating tumor DNA (ctDNA) may be a useful tool to identify tumor molecular dynamics, improving the personalization of treatments for mBC patients [15,16]. In the present study, the clinical outcomes of hormone therapy and CDK4/6i in mBC patients are investigated on the basis of the presence of ESR1 mutations as analyzed by liquid biopsy.

## 2. Materials and Methods

### 2.1. Patients and Data Collection

The present retrospective pharmacogenetic study looked at mBC patients treated with palbociclib/ribociclib/abemaciclib as first- or second-line therapy in association with hormonal therapy (letrozole or fulvestrant) as per approved label. Patients may have been treated with adjuvant endocrine therapy with aromatase inhibitor or tamoxifen as per clinical practice. According to the duration of previous endocrine response, each patient was classified as endocrine-sensitive (if relapsed at least 12 months after the completion of adjuvant endocrine therapy or with de novo advanced breast cancer) or endocrine-resistant (if relapsed within 12 months after ending adjuvant endocrine therapy). Clinical parameters, such as complete response (CR), partial response (PR), stable disease (SD), and progressive disease (PD), were defined following RECIST (v. 1.1) criteria.

### 2.2. Circulating Free DNA Extraction and ESR1 Mutational Analysis

Twelve ml of blood was collected at baseline (prior to CDK4/6i) in EDTA tubes and centrifuged at 1900× *g* for 10 min at 4 °C within 2 h after drawing to collect plasma, which was stored at −80 °C until analysis. Plasma samples were centrifuged again at 1900× *g* for 15 min to remove cellular debris. Circulating free DNA (cfDNA) extraction was performed using an Avenio cfDNA isolation kit (Roche, Basel, Switzerland). cfDNA analysis of ESR1 mutations was performed by QX200 ddPCR (Bio-Rad, Hercules, CA, USA) using ddPCR ESR1 Multiplex Assay 1 (dHsaMDXE91450042) and 2 (dHsaMDXE91450042, dHsaMDXE65719815) for human ESR1. Multiplex 1 contained FAM-labeled probes for p.E380Q (c.1138G > C), p.L536R (c.1607T > G), p.Y537C (c.1610A > G), and p.D538G (c.1613A > G). Multiplex 2 contained FAM-labeled probes for p.S463P (c.1387T > C), p.Y537N (c.1609T > A), and p.Y537S (c.1610A > C). Fluorescence signal quantification was performed using a droplet reader and QuantaSoft software (Bio-Rad, Hercules, CA). Droplets with a fluorescence intensity threshold higher than 4000 were considered positive, and the amount was expressed as copies/mL.

### 2.3. Statistical Analysis

Categorical variables, such as stage at diagnosis, pre-/post-menopause, ECOG performance status, number and sites of metastasis (i.e., presence of visceral disease), CDK4/6i line of therapy, type of associated hormonotherapy (HT) to CDK4/6i, previous chemotherapy (CT) in the adjuvant and metastatic setting, previous HT in the adjuvant and metastatic setting, ESR1 mutation, PR and Ki67 intervals (according to the 2011 and the 2013 St. Gallen criteria) [17,18,19], and patient clinical outcome, were described by absolute and relative frequencies. Quantitative factors such as age, ER, PR, and Ki67 expression in primary lesions were assessed by mean ± standard deviation (STD). Disease-free survival (DFS) was defined as the length of time from the start of hormonal adjuvant treatment to relapse, while progression-free (PFS) and overall survivals (OS) were defined as the time from start of treatment to PD or death from any cause. DFS, PFS, and OS curves were illustrated using Kaplan–Meier analyses and log-rank tests, and Cox proportional hazard models evaluated hazard ratio (HR) and 95% confidence interval (CI). An χ2-test was used to determine whether any associations between ESR1 somatic mutation and the other categorical variables in the sample was likely to reflect a real association in the population. Differences were considered significant at *p* < 0.05. All statistical calculations were performed with MedCalc Statistical Software version 14.8.1 (MedCalc Software bvba, Ostend, Belgium), URL, http://www.medcalc.org (accessed on 15 November 2022).

## 3. Results

### 3.1. Patient Characteristics

Forty-two patients were enrolled in the study. Clinical data of patients are reported in Table 1. Of 42 patients, 37 (81.1%) were ER+/PR+, and 5 (11.9%) were ER+/PR−. In particular, 4 patients (9.5%) and 38 patients (90.5%) had an ER% expressions of 51–80% and 81–100%, respectively. Moreover, 9 patients (21.4%), 3 patients (7.1%), 8 patients (19.1%), and 22 patients (52.4%) had a PR% of 0–20%, 21–50%, 51–80%, and 81–100%, respectively.

Premenopausal patients received triptorelin in association with exemestane. In the adjuvant setting, 28 patients received tamoxifen (*n* = 11) and AI (*n* = 17) as hormonal therapy, with a median DFS of 60 months. Overall, patients received palbociclib (*n* = 15), ribociclib (*n* = 20), or abemaciclib (*n* = 7) plus hormonal therapy (letrozole or fulvestrant) for metastatic disease treatment. Median follow-up was 12 months.

Thirty-five patients were treated with CDK4/6i as first-line treatment as follows: 10 patients were treated with palbociclib, 20 received ribociclib, and 5 abemaciclib. Seven patients received palbociclib (*n* = 5) and abemaciclib (*n* = 2) as second-line therapy. In latter group of patients, chemotherapy was the main first-line treatment, including capecitabine plus vinorelbine (*n* = 2), taxol plus bevacizumab (*n* = 3), while two patients received exemestane alone. Considering the overall population that received CDK4/6i as first- or second-line treatment, the median OS was 49.8 months. Among patients who received CDK4/6i plus hormonal therapy as first-line treatment, the median PFS to CDK4/6i was not reached. Twenty-six patients were classified as endocrine-sensitive, and nine were endocrine-resistant. A total of 25 patients had ≤2 metastatic sites, and 10 patients had >2 metastatic sites. Six patients had bone-only disease.

### 3.2. ESR1 Mutation Is an Independent Predictive Biomarker of Clinical Recurrence after Adjuvant Therapy

**Figure 1:**
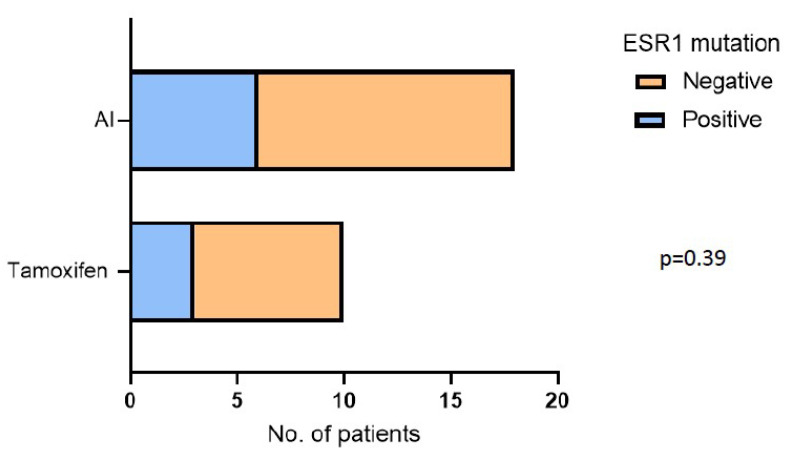
Incidence of ESR1 mutations and their association with the type of hormonal therapy received. AI: aromatase inhibitor.

Among patients who received the adjuvant treatment, nine (32%) presented with an ESR1 mutation; in particular, two patients were carriers of p.D538G, four harbored p.Y537C, two showed p.E380Q, and one patient had the p.L536R mutation. Moreover, three patients (30%) treated with tamoxifen and six patients (50%) treated with AI presented an ESR1 mutation, as reported in Figure 1.

**Figure 2:**
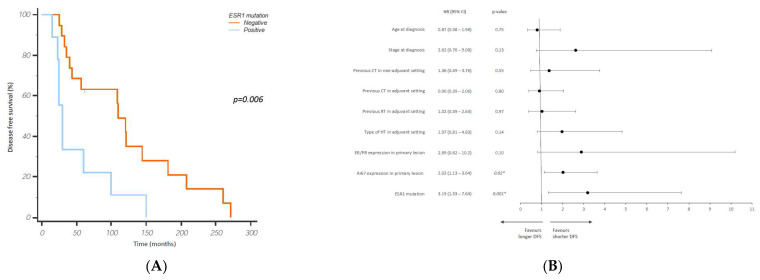
Disease-free survival (**A**) of breast cancer patients treated with adjuvant hormonal therapy. Univariate Cox regression analysis (**B**) of the impact of ESR1 mutational status and patients’ clinical characteristics on DFS. * *p* < 0.05.

No statistical differences between the presence of ESR1 mutation and the type of adjuvant hormonal therapy were found (*p* = 0.39). Patients harboring an ESR1 mutation in blood at disease recurrence (first-line therapy, baseline) had a significantly shorter DFS compared to patients without ESR1 mutations (30 vs. 110 months; 9 vs. 19 patients; *p* = 0.006; Figure 2A). Univariate and multivariate Cox regression analyses were performed to assess the effect of ESR1 mutation status on the prediction of time-to-event outcomes (Figure 2B).

The DFS univariate model showed an association between the presence of ESR1 mutation (HR = 3.18; 95% CI = 1.33–7.64; *p* = 0.009) and KI67 expression level (HR = 2.02; 95% CI = 1.13–3.64; *p* = 0.02). In multivariate analysis, ESR1 mutations were confirmed as independent factors of resistance to adjuvant hormonal therapy (HR = 3.54; 95% CI = 1.19–10.52; *p* = 0.02).

### 3.3. CDK4/6i Overcomes Hormone Therapy Resistance in ESR1 Mutant Patients

**Figure 3:**
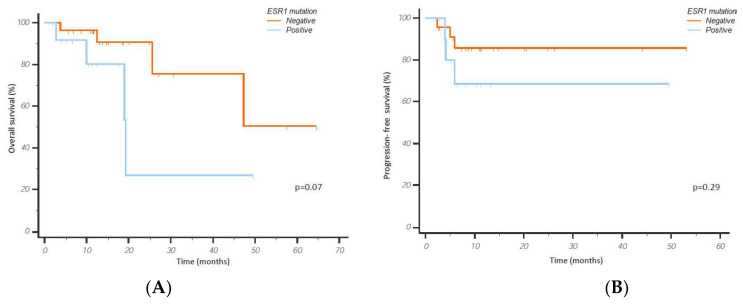
Overall survival of mBC patients treated with CDK4/6i (**A**) and progression-free survival of first-line CDK4/6i-treated patients (**B**) according to ESR1 mutational status.

Among patients who received CDK4/6i in the metastatic setting, 13 (31%) presented an ESR1 mutation at baseline. Considering the overall population, the median OS was 19.3 months for ESR1 mutant patients vs. not reached in patients without ESR1 mutations (13 vs. 29 patients; *p* = 0.07; Figure 3A). Among patients who received CDK4/6i as first-line treatment, the median PFS was calculated. No statistically significant differences in terms of PFS were found comparing ESR1 mutant and non-mutant patients (not reached in either group; 12 vs. 23 patients; *p* = 0.29; Figure 3B).

**Figure 4:**
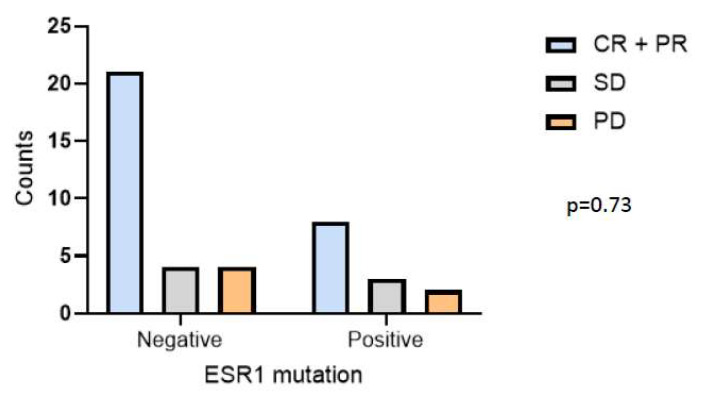
Incidence of ESR1 mutations and their association with best response of patients in the overall population.

According to the best response, patients were divided into two groups; 29 patients (69.04%) were identified to have a complete or partial response (CR/PR), whereas 13 patients (30.96%) presented as stable or with a progression disease (SD/PD). The objective clinical benefit among all patients enrolled was 61.9%. Among the CR/PR group, 21 patients (72.41%) carried the ESR1 mutation, whereas 8 patients (27.59%) were wild-type. Moreover, among the SD group, three patients (42.90%) carried the ESR1 mutation, while four (47.10%) did not. Concerning the PD group, two patients (33.33%) carried the ESR1 mutation, while four (66.67%) did not. The association between ESR1 mutational status and the clinical response (CR/PR or SD or PD) was not statistically significant (*p* = 0.73; Figure 4).

## 4. Discussion

The present study examined the association between ESR1 mutational status and the response to hormonal therapy and CDK4/6 inhibitors. While ESR1 mutations were found to have a negative predictive role for DFS after adjuvant treatment, no association was found with CDK4/6i first-line treatment outcome, highlighting the role of CDK4/6i potential to overcome ESR1-dependent resistance. Moreover, no statistically significant association between ESR1 mutational status and response (CR/PR or SD/PD) was found.

Previous studies reported no significant impact of ESR1 mutations on PFS in patients treated with fulvestrant alone or in combination with CDK4/6i [20,21,22]. However, ESR1 drives tumor cell growth and proliferation, and its upregulation or the appearance of activating mutations may be responsible for resistance to hormonal treatments [23,24]. In fact, several studies displayed a correlation between the presence of mutations in the ESR1 receptor and the acquisition of endocrine resistance in a large percentage of mBC patients [5,11,25,26,27].

Accordingly, the present study demonstrates that patients harboring an ESR1 mutation at disease recurrence have a significantly shorter DFS compared to patients without mutations (30 vs. 110 months, *p* = 0.006). This was also demonstrated with Cox regression analysis, which compared the presence of ESR1 mutations with clinical characteristics such as age, previous neoadjuvant or adjuvant therapies, ER or PR expression, and mitotic index (Ki67) in primary cancer. Importantly, the presence of an ESR1 mutation as an independent predictive factor of clinical recurrence was maintained in the multivariate analysis. This result is consistent with many other clinical studies, demonstrating the crucial role of ESR1 mutation as a driver of resistance and worse outcome in metastatic breast cancer patients treated with aromatase inhibitors (AI), suggesting also that ESR1 mutations could be detected soon as the first relapse to guide pharmacological intervention [12,28,29,30,31].

It is known that AIs do not bind directly to estrogen receptors; however, they are able to reduce the levels of the estrogen ligand [32]. Moreover, Jeselsohon et al. demonstrated that mutations in the LBD of the ESR1 confer partial resistance to tamoxifen (or fulvestrant), probably due to a conformational change of the ER, leading to a decreased drug affinity [33]. Results from the PADA-1 trial showed that the presence of ESR1 mutations in liquid biopsy at the baseline of the first-line treatment may be a predictive marker for patients treated with AI + palbociclib; however, the frequent clearance of the ERS1 mutation during AI + palbociclib treatment (often after the first cycle) suggests that AI + palbociclib retains some activity despite ESR1 mutations. The PADA-1 trial was the first to demonstrate the clinical utility of ESR1 mutations, showing that ESR1 mutation monitoring in liquid biopsy allows for optimization the endocrine therapy partner of CDK4/6i, and upon ESR1 mutation detection, the mPFS was doubled by the switch from AI–palbociclib to fulvestrant–palbociclib. Therefore, the PADA-1 trial highlights that the implementation of the PADA-1 treatment strategy may be a valid option in mBC routine care, as well as the need for the development of new SERDs for mBC patients [13,14]. One of the limitations of the present study is that the timing of ESR1 mutation appearance is unknown. Moreover, considering tumor heterogeneity and the small size of our population, looking for ESR1 mutations may not be sufficient, since other mutations, such as in the MAPK, PI3K/AKT/mTOR, and CDK4/6 pathways, have been demonstrated to be involved in the mechanisms of resistance [7,34]. Recently, studies on liquid biopsy demonstrated the subclonal origin of different mutations (i.e., ESR1) in pretreated advanced ER + breast cancer and their implication for response to therapy [35,36].

In the plasmaMATCH trial, ctDNA sequencing was used to interrogate the genomic profile of 800 advanced breast cancer patients. The authors demonstrated the copresence of different subclonal resistance mutations, in particular mutations in the ESR1 and MAPK pathways and their association with poor overall survival [35]. Similarly, Sivakumar et al. showed the tumor evolution landscape of breast cancer, identifying higher frequencies of polyclonal acquired alterations associated with resistance to endocrine therapy [36].

Results from the PALOMA-3 study highlighted the involvement of PI3K, AKT, RB1, E2F, or CCNE1 as intrinsic and acquired mechanisms of resistance to CDK4/6i [20,21,37,38,39]. Additionally, several potential mechanisms, such as loss of ER expression, increased expression of ER or its cofactors, and post-translational ER modifications including methylation, acetylation, and SUMOylation, have been studied in the context of HR-positive mBC sensitivity to therapies [40,41]. In addition, the delocalization of the ER to the cellular membrane, enabling ER crosstalk with other proteins, including growth factor receptors, is involved in the development of the endocrine-resistant phenotype [42].

Despite effective hormonal and CDK4/6i treatment, the development of new additional targeted therapies is needed to prolong the survival of patients [43]. Recently, new estrogen receptor antagonists (i.e., lasoxifene, bazedoxifene, amcenestrant, camizestrant, and elacestrant) have been developed and evaluated in preclinical and clinical studies alone or in combination with CDK4/6i [13,44,45,46,47,48,49,50,51,52], demonstrating their superiority to fulvestrant, especially for patients harboring ESR1 mutations.

Hence, it is necessity to discover new drugs and biomarkers to identify patients who may or may not respond to specific treatments, thereby improving their long-term survival. From the perspective of future precision medicine, liquid biopsy investigation is advantageous, owing to its low invasiveness and high potential to monitor and support the best therapeutic choice for patients.

## 5. Conclusions

The present study demonstrates that CDK4/6 inhibition in combination with either aromatase inhibitors or fulvestrant does not preclude hormonal therapy failure caused by ESR1 mutations.

## Figures and Tables

**Table 1:** Characteristics of patients.

Characteristics	Patients (*n* = 42)
Age at diagnosis, median (range)	55.1 ± 11.3
Stage at diagnosis, *n* (%) I II III IV (i.e., de novo M1)	14 (33.3%) 11 (26.2%) 6 (14.3%) 11 (26.2%)
ECOG PS, *n* (%) 0 1	38 (90.5%) 4 (9.5%)
Pre/Post-menopause, *n* (%) Pre-menopause Post-menopause	6 (14.3%) 36 (85.7%)
HR receptor status in primitive lesions, *n* (%) ER+/PR+ ER+/PR−	37 (88.1%) 5 (11.9%)
Ki67 % in primitive lesions, *n* (%) <14%, *n* (%) 14–20%, *n* (%) >20%, *n* (%)	17 (40.5%) 8 (19%) 17 (40.5%)
Previous CT in neoadjuvant setting, *n* (%) No Yes	36 (85.7%) 6 (14.3%)
Previous CT in adjuvant setting, *n* (%) No Yes	24 (57.1%) 18 (42.9%)
Previous HT in adjuvant setting, *n* (%) No Yes	14 (33.3%) 28 (66.7%)
Previous RT in adjuvant setting, *n* (%) No Yes	20 (47.6%) 22 (52.4%)
Number of metastatic sites, *n* (%) ≤2 >2	28 (66.7%) 14 (33.3%)
Disease site, *n* (%) Visceral Bone-only Nodes	22 (52.4%) 6 (14.3%) 14 (33.3%)
Endocrine-sensitive or resistant disease, *n* (%) Sensitive Resistant	29 (69%) 13 (31%)
CDK4/6i therapy, *n* (%) Palbociclib Ribociclib Abemaciclib	15 (35.7%) 20 (47.6%) 7 (16.7%)
CDK4/6i line of therapy, *n* (%) 1 2	35 (83.3%) 7 (16.7%)
Type of HT associated to CDK4/6i, *n* (%) Letrozole Fulvestrant	25 (59.5%) 17 (40.5%)

## Data Availability

The data presented in this study are available in this article.
